# Automated synapse-level reconstruction of neural circuits in the larval zebrafish brain

**DOI:** 10.1038/s41592-022-01621-0

**Published:** 2022-10-24

**Authors:** Fabian Svara, Dominique Förster, Fumi Kubo, Michał Januszewski, Marco dal Maschio, Philipp J. Schubert, Jörgen Kornfeld, Adrian A. Wanner, Eva Laurell, Winfried Denk, Herwig Baier

**Affiliations:** 1 Max Planck Institute for Biological Intelligence, Martinsried, Germany; 2 Max Planck Institute for Neurobiology of Behavior - caesar, Bonn, Germany; 3 Center for Frontier Research, National Institute of Genetics, Mishima, Japan; 4 Google Research, Zürich, Switzerland; 5 Department of Biomedical Sciences, University of Padova, Padova, Italy; 6 Princeton Neuroscience Institute, Princeton University, Princeton, NJ USA; 7 Paul Scherrer Institute (PSI), Villigen, Switzerland; 8 Present Address: ariadne.ai ag, Buchrain, Switzerland; 9 Present Address: Department of Neurology, Faculty of Medicine and University Hospital Cologne, University of Cologne, Cologne, Germany

**Keywords:** Neuroscience, Software, Zebrafish, Scanning electron microscopy

## Abstract

Dense reconstruction of synaptic connectivity requires high-resolution electron microscopy images of entire brains and tools to efficiently trace neuronal wires across the volume. To generate such a resource, we sectioned and imaged a larval zebrafish brain by serial block-face electron microscopy at a voxel size of 14 × 14 × 25 nm^3^. We segmented the resulting dataset with the flood-filling network algorithm, automated the detection of chemical synapses and validated the results by comparisons to transmission electron microscopic images and light-microscopic reconstructions. Neurons and their connections are stored in the form of a queryable and expandable digital address book. We reconstructed a network of 208 neurons involved in visual motion processing, most of them located in the pretectum, which had been functionally characterized in the same specimen by two-photon calcium imaging. Moreover, we mapped all 407 presynaptic and postsynaptic partners of two superficial interneurons in the tectum. The resource developed here serves as a foundation for synaptic-resolution circuit analyses in the zebrafish nervous system.

## Abstract (web summary)

This Resource presents a serial block-face EM dataset of the whole larval zebrafish brain, including automated segmentation of neurons, detection of synapses and reconstruction of circuitry for visual motion processing.

## Main

Information processing in the central nervous system is carried out by interconnected neurons. Synapses, the key sites of communication in this network, can only be identified and assigned to a specific pair of neurons with electron microscopy (EM). The first attempt to reconstruct the nervous system wiring of an organism, the nematode worm *Caenorhabditis elegans*^1^, created a connectivity map that was essential for the study of neural circuits^2^. In the meantime, EM reconstructions of the brain have been generated for invertebrate species, including larval^3^ and adult *Drosophila melanogaster*^4,5^ and the annelid *Platynereis dumerilii*^6^. Until now, whole-brain datasets of comparable resolution have not been made available for a vertebrate.

Tying function to structure at synaptic resolution may involve recording of neuronal activity, for example, by two-photon (2P) calcium imaging, followed by reconstruction of the underlying cellular connectivity with volume EM (vEM)^7–11^.

**Fig. 1:**
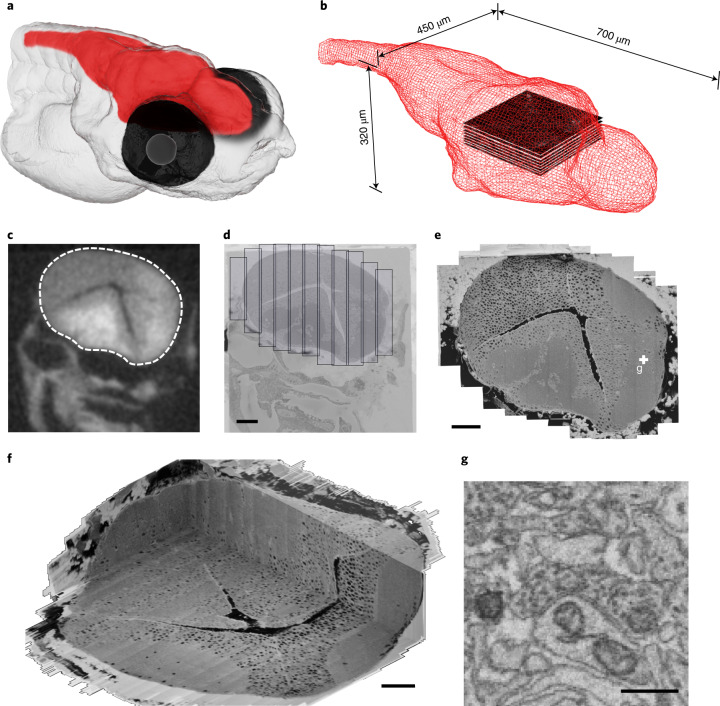
Pretectal 2P calcium imaging and whole-brain larval SBEM dataset acquisition. **a**, Illustrative larval zebrafish head at 5 dpf, brain highlighted in red. **b**, Wire-frame representation of a 5 dpf zebrafish brain, slice stack represents location of the 2P calcium imaged volume centered on the pretectum. **c**,**d**, X-ray image (**c**) of sample used to calculate horizontally stacked tile pattern (**d**), shown here overlaid on a low-resolution vEM overview image. **e**, High-resolution vEM imaged brain slice stitched from individual tiles shown in **d**. **f**, The resulting high-resolution volume. **g**, Synaptic contact in pretectum, as highlighted in **e**. Scale bars, 50 μm (**d**–**f**), 500 nm (**g**).

The larval zebrafish (*Danio rerio*) is uniquely suited to this approach^11,12^. Work in the zebrafish olfactory bulb has revealed fundamental principles of odor processing^11,13^. At 5 days postfertilization (dpf), the larval brain is comparable in size to that of adult *Drosophila*: 700 µm from rostral (tip of the olfactory bulb) to caudal (commissura infima of Haller), maximally 450 µm wide (at the level of the midbrain) and maximally 320 µm from dorsal to ventral surface^14^ (Fig. 1a,b). Brain-wide catalogs of thousands of single-neuron morphologies and area-to-area (mesoscale) wiring diagrams have been generated for zebrafish larvae^15^. These data offer a plausibility check on vEM tracings, especially of long-range projections.

While the benefits of a connectomic approach have become widely accepted, the tools and resources needed are only available to a small set of laboratories and/or do not have the required resolution for synapse-scale reconstructions^16^. Storage, handling and sharing of large amounts of data require expensive hardware and software, posing large challenges for individual laboratories. Also, manual reconstructions of connectivity are labor-intensive and costly. Thus, with the exception of the olfactory bulb work mentioned above^11,13^, EM-based circuit analysis in zebrafish has been limited to few cells in specific areas of the nervous system^10,17–21^. One of our aims is to make connectomic data and computational tools available to the entire zebrafish community.

Here we provide access to an EM dataset covering the entire zebrafish brain (5 dpf), except for the retinae, allowing the identification of chemical synapses and tracing in all directions. We acquired images with serial block-face scanning EM (SBEM) at 14 × 14 × 25 nm^3^ voxel size. We reconstructed and proofread several hundred visual motion-processing neurons previously identified in the same specimen by in vivo 2P calcium imaging. Cells and wires were segmented by flood-filling networks (FFNs)^22^. We applied a synapse detection algorithm to the entire volume and automatically generated a brain-wide map of synaptic connections. We also introduce a digital address book of neurons and synapses, linked to the spatial coordinates of the volume. To demonstrate broad use of the computational pipeline, we mapped all 407 input and output cells for two tectal superficial interneurons (SINs), which led to the identification of an interconnected SIN network. The resource presented here, together with the computational framework, promises to offer detailed biological insights into zebrafish neural circuits.

## Results

### Functional imaging of motion-processing neurons

Using 2P microscopy, we recorded neuronal activity from a cuboid of the larval zebrafish brain. This cuboid contains motion-processing neurons and includes the pretectum and adjacent parts of the tectum, tegmentum and thalamus. Pretectal areas are involved in the processing of optic flow^23–31^. The fish larva expressed the genetically encoded Ca^2+^ indicator GCaMP5G in most neurons and was exposed to a succession of eight drifting grating stimuli, designed to cover all combinations of horizontal optic flow^25^.

Individual motion-sensitive neurons responded differently from one, or a combination, of the eight motion phases. In a single brain, we typically observe 200 to 300 neurons whose activity is well-predicted by one of the 255 theoretically possible response profiles^25^. The individual fish we chose for vEM imaging had a total of 216 functionally annotated motion-sensitive cells, each falling into one of the 11 most frequently observed response types (4–30 cells per type).

### X-ray tomography-targeted SBEM imaging

**Extended Data Fig. 1:**
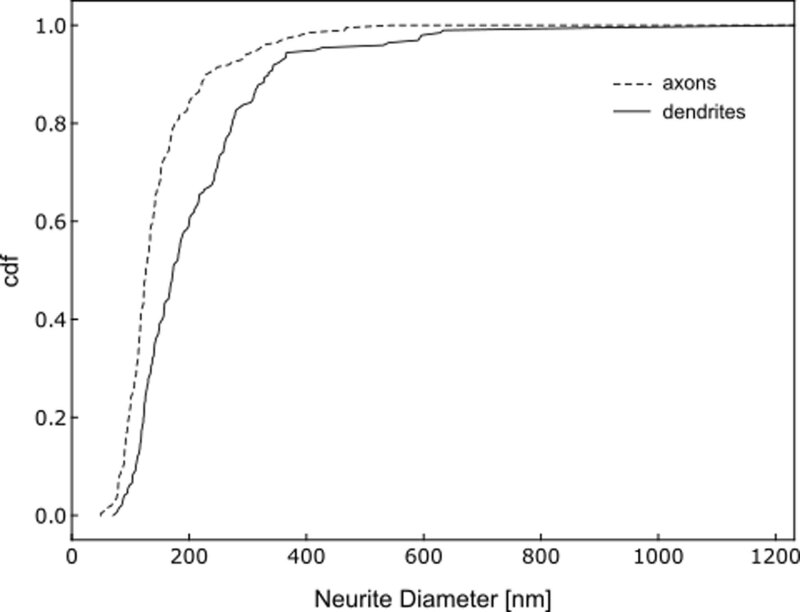
Neurite diameter distribution. Neurite diameter distribution for pretectal cells (cumulative density function, cdf), measured perpendicular to the main neurite axis in axons and dendrites, at 200 randomly sampled locations in each.

The acquisition time needed to image a given volume rises sharply as the resolution is increased. The structures we need to reliably identify in our image stack are neurites (as thin as 50 nm), synaptic vesicles (around 40 nm in diameter) and postsynaptic densities (25–50 nm thick) (Extended Data Fig. 1). These lengths therefore give a lower bound for acceptable resolution. We chose a section thickness of 25 nm and a pixel size of 14 × 14 nm^2^. This scale allows us to resolve even the thinnest wires and detect features of chemical synapses.

To minimize acquisition time, we optimized two steps. First, we developed X-ray guided adaptive tiling, where we used a micro-computed tomography (micro-CT) scan of the embedded sample registered to the SBEM microtome stage coordinate system to automatically target SBEM imaging to only the brain, avoiding the surrounding embedding material and other parts of the head (Fig. 1c–g). Second, we used piezo scanning along the slow axis, removing field of view (FOV) limitations along that axis and reducing the number of motor moves required to tile each section. The combination of X-ray targeting and piezo scanning reduced the total acquisition time from an estimated 160 to 66 days.

### Tracing of ‘optic-flow’ neurons and their connectivity

**Fig. 2:**
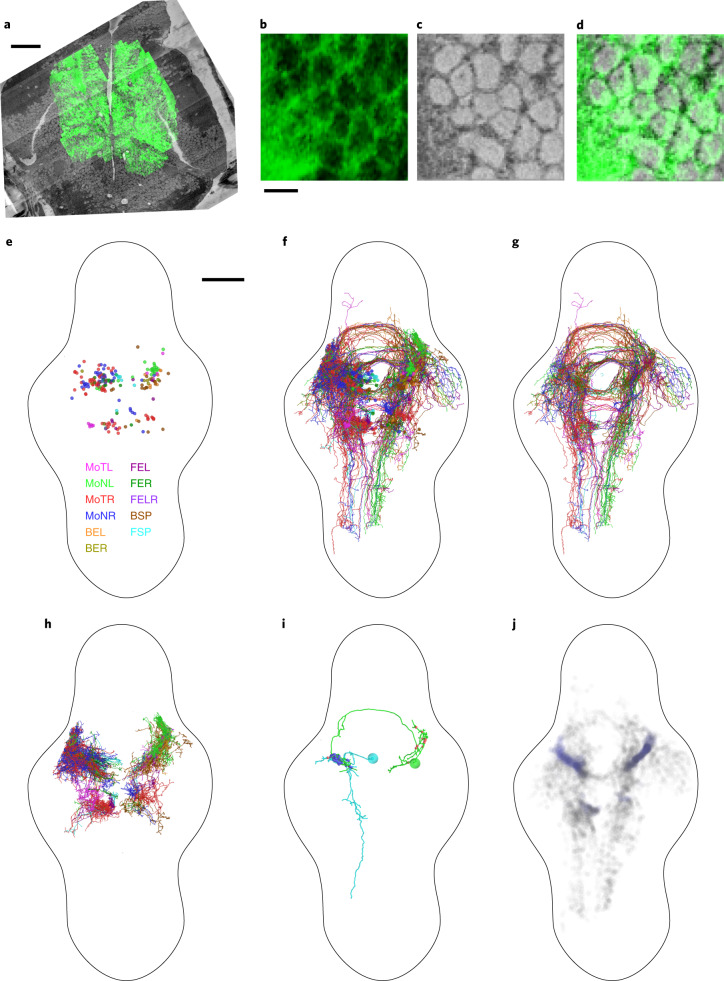
Mapping and EM-based reconstruction of functionally characterized pretectal neurons. **a**, Single plane of GCaMP5G fluorescence registered to SBEM dataset. **b**–**d**, Zoomed view of data in **a**, showing GCaMP5G (**b**) and scanning EM image (**c**) individually and as overlay (**d**). **e**–**i**, Panels show tracings seeded from soma centers (*n* = 208) (**e**), with functional response types classified and named as in ref. ^25^, neurons skeletonized from those seeds (**f**), axons (**g**), dendrites (**h**) and two individual example neurons (**i**), for all of the functionally characterized, EM-reconstructed cells, colored by functional response type. Blue and red spheres in **i** indicate incoming and outgoing synapse locations, respectively. **j**, All synapse locations with traced (blue) and untraced (black) postsynaptic partners. MoNL, MoNR, MoTL and MoTR refer to monocular nasalward (N) or temporalward (T), left eye (L) or right eye (R). FEL, FER, BEL, BER refers to forward- (F) or backward- (B) selective, excited by left (L) or right (R) eye. FELR refers to forward-selective, excited by left and right eye. FSP and BSP refer to forward (F) or backward (B) specific. Scale bars, 50 μm (**a**), 5 μm (**b**–**d**) and 100 μm (**e**–**j**).

To establish the correspondence between functionally identified cells and cells in the vEM dataset, we aligned a high-resolution 2P *z*-stack, taken after the functional recordings, to the vEM stack coordinates (Fig. 2a–d) by block-wise affine registration. We iteratively selected correspondence points, proceeding from large-scale structures (ventricles and blood vessels) to finer ones (for example, individual somata).

**Extended Data Fig. 2:**
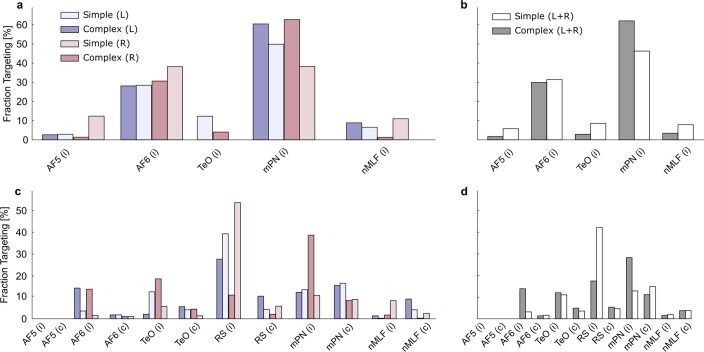
Between-hemisphere comparison of reconstructed pretectal cell morphology. Fraction of skeleton branch points, taken over all cells, present in different anatomical areas for dendrites of simple (MoNL, MoNR, MoTL and MoTR) and complex (FEL, FER, BEL, BER, FELR, FSP and BSP) cells (complex left: 38, right: 36; simple left: 94, right: 40) in the left (L) and right (R) hemisphere separately (**a**) and in both hemispheres (**b**). **c**-**d**. Like **a**-**b**, for the axons. AF5, AF6: Retinal arborization fields 5 and 6, TeO: optic tectum, mPN: medial pretectal neuropil, nMLF: neuropil of the nucleus of the medial longitudinal fasciculus, vHb: ventral hindbrain neuropil. (i) and (c) indicate ipsilateral and contralateral, respectively. AF and nMLF annotations are based on LM atlas masks registered to the EM data.

**Extended Data Fig. 3:**
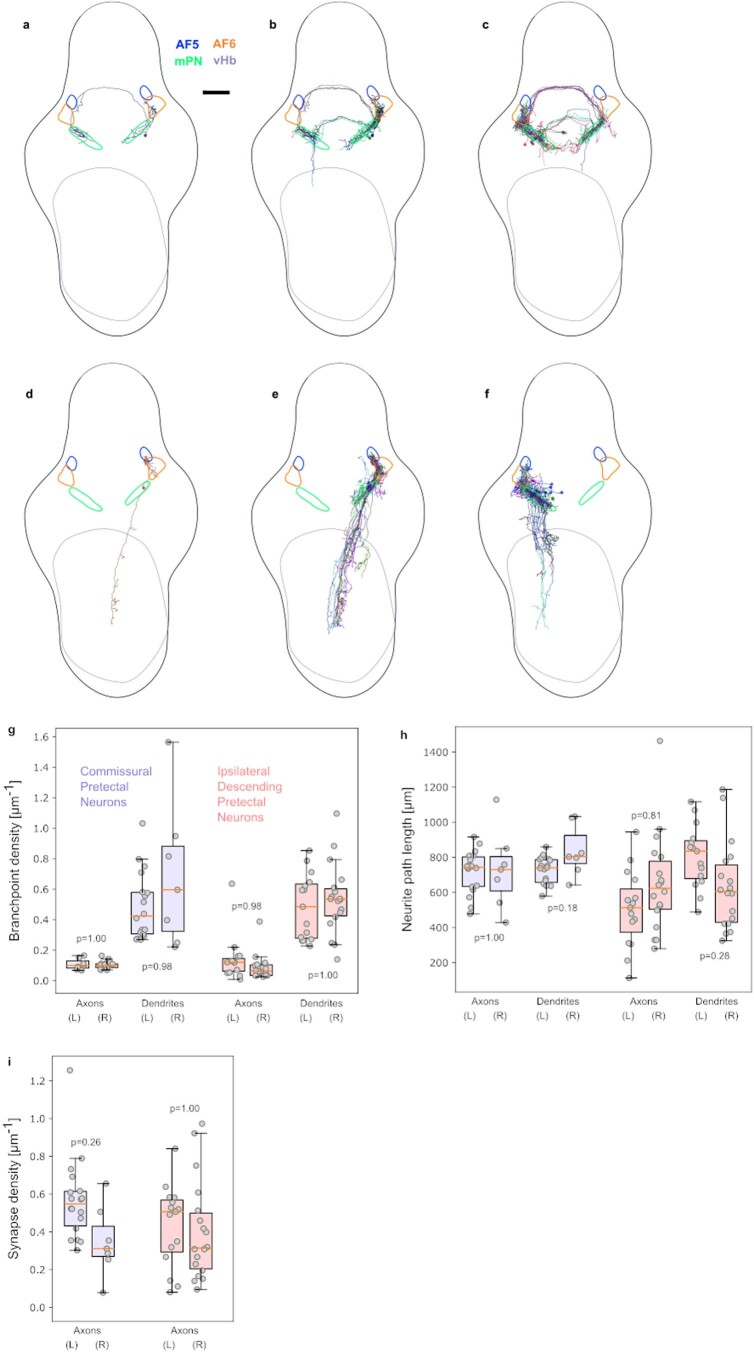
Bilaterally symmetric reconstructions of two morphologically defined pretectal cell types. Individual example of a commissural pretectal interneuron (**a**) and all neurons of this type with somata on the right (**b**) and left (**c**) side of the brain. **d**-**f**. Like **a-c**, for ipsilateral descending pretectal projection neurons. **g**-**i**. Branchpoint densities (**g**), neurite path lengths (**h**) and axonal synapse densities (**i**) for commissural pretectal interneurons (blue, n = 18 and 7 cells on the left and right side, respectively) and ipsilateral descending pretectal projection neurons (red, n = 15 and 18 cells on the left and right side, respectively), plotted separately for axons and dendrites and for the two brain hemispheres. AF5, AF6: Retinal arborization fields 5 and 6, mPN: medial pretectal neuropil region, vHb: ventral hindbrain neuropil. AF annotations are based on LM atlas masks registered to the EM data. Box plot center lines represent medians, box limits upper and lower quartiles, whiskers 1.5x the interquartile range. Scale bar: 100 μm.

For 208 of the 216 cells characterized by function, we could unambiguously identify the corresponding somata in the vEM dataset. We manually skeletonized these neurons (Fig. 2e–h and Extended Data Figs. 2 and 3) using the CORE annotation procedure^13^. This yielded a total neurite path length of 280.8 mm, of which 139.5 mm were dendrites and 141.3 mm axons (1,350.0 ± 556.7, 670.8 ± 222.4 and 679.2 ± 422.7 mean ± s.d. µm per cell, respectively). Subsequently, we identified synapses between the reconstructed cells, yielding a total of 1,079 synaptic contacts among these 208 neurons (Fig. 2i,j).

Many of the axons and dendrites of our reconstructed neurons contacted an area of pretectal neuropil, which we provisionally named the medial pretectal neuropil (mPN, corresponding to the region of high synapse density in Fig. 2j; also Extended Data Figs. 2 and 3). Twenty-five of our 208 optic-flow responsive neurons had dendrites in ipsilateral retinorecipient neuropil areas^32^ (AF5, AF6) and/or mPN and, through the posterior or postoptic commissures, projected axons to the contralateral mPN and/or AF6 (Fig. 2i). Another common cell type (33 out of 208 cells) had dendrites in AF5, AF6 or mPN, but the axon coursed ventrally and caudally into the ipsilateral ventral hindbrain. As an internal control to assess reproducibility of reconstructions in this vEM dataset, we quantified path length, branchpoint density and axonal synapse density for these two cell types in both hemispheres separately (Extended Data Fig. 3). No interhemisphere comparisons were statistically significantly different (*P* values ranged from 0.18 to 1.00 and are shown for each comparison in Extended Data Fig. 3; independent two-sample *t*-tests with Holm–Bonferroni correction). We will publish a detailed analysis of the pretectal connectivity elsewhere.

**Fig. 3:**
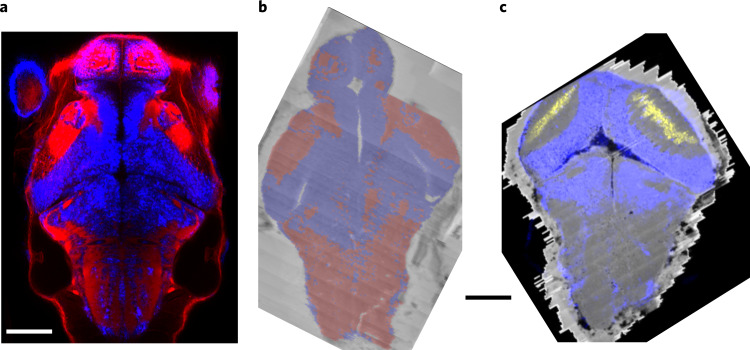
Registration of LM atlas dataset and vEM stack. **a**, *elavl3:lynTag-RFP* (red) and *elavl3:H2B-GCaMP6s* (false-colored in blue) fluorescence stacks, registered into a common coordinate system. Dorsal view. **b**, Soma (blue) and neuropil (red) prediction on low-resolution overview vEM data used as registration target, overlaid over raw data. **c**. Overlay of *elavl3:H2B-GCaMP6s* (blue) and *pou4f3:mGFP* (yellow) registered into the vEM brain coordinate system and shown over full resolution vEM data. Scale bars, 100 μm.

**Extended Data Fig. 4:**
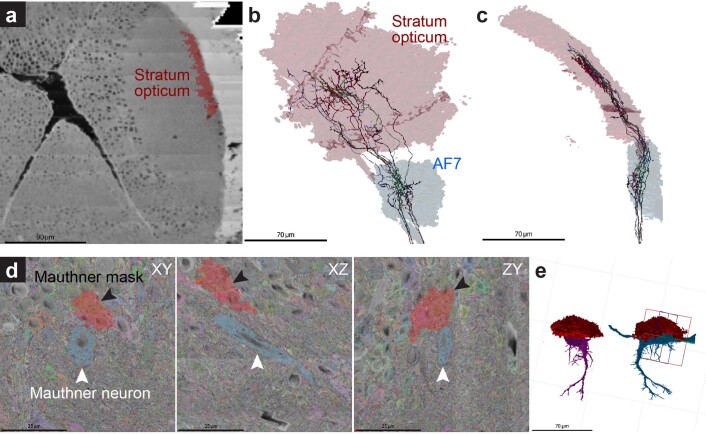
LM-to-EM registration match precision. Examples for the accuracy of registrations. **a**-**c**: Retinal ganglion cell axons (n = 7), which have been traced in the EM dataset and which project both to the tectal stratum opticum (SO) and the arborization field 7 (AF7), localize exclusively inside the registered mapzebrain regions. **d**-**e**: The registered mask for the Mauthner neurons, which was generated in the mapzebrain atlas (red, black arrowheads), deviates by ~20 µm from the location of the Mauthner cells in the EM dataset (blue, white arrowheads). Second Mauthner cell is shown in magenta in **e**.

Finally, we registered the Max Planck Zebrafish Brain Atlas (mapzebrain, http://mapzebrain.org) to our vEM dataset. The mapzebrain atlas offers annotated masks of 112 areas in a reference larval brain (‘standard brain’). We performed the mapping based on the distribution of soma and neuropil areas in both datasets (Fig. 3a,b). This brought the EM and light microscopy (LM) coordinate systems into register (Fig. 3c) with a deviation of maximally 20 µm (Extended Data Fig. 4).

### Automated segmentation and human proofreading

We trained three different three-dimensional (3D) CNNs to perform semantic segmentation of the EM volume. Model 1 classified voxels into neuropil, somata or a class that includes both blood vessels and ventricles. Model 2 classified voxels into ‘neuropil’ or ‘not-neuropil’. Model 3 performed a classification into ‘nucleus’ or ‘not-nucleus’. We then applied a size filter of 1,000 voxels, yielding 121,956 putative nuclei.

**Fig. 4:**
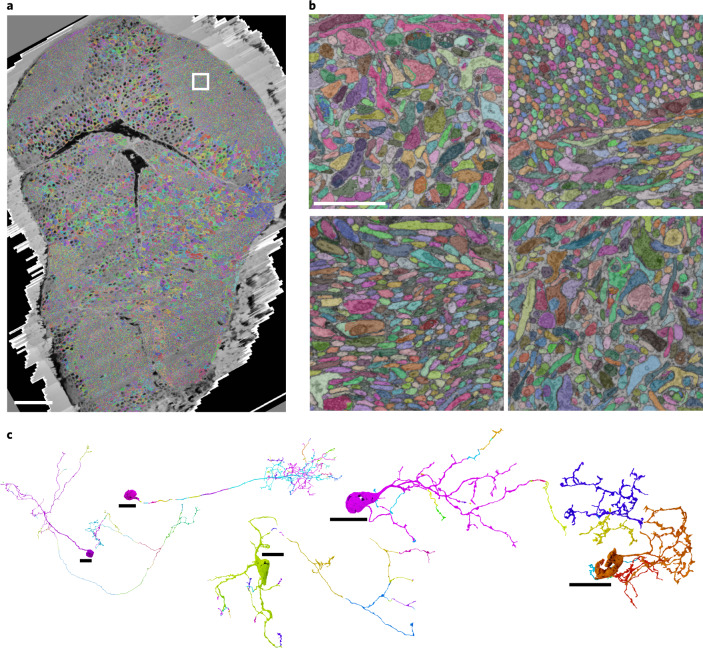
Automated neurite segmentation. **a**, Dorsal view of the vEM dataset with a multicolored overlay of the base segmentation. **b**, Close-up examples (one out of at least ten) of the segmentation in, from top left to bottom right, the tectal neuropil (highlighted in **a**), rostral hypothalamus, intermediate and inferior ventral medulla oblongata. **c**, Examples of semiautomatically reconstructed neurons. Numbers of corrected merge and split errors are given in parentheses. From left to right: dorsal thalamic projection neuron (mergers, 1; splits, 62), tectal PVIN (1, 124), inferior raphe neuron (14, 52), inferior dorsal medulla oblongata neuron (0, 23) and pretectal interneuron (0, 8). Different colors indicate neuron fragments merged manually. Scale bars, 50 µm (**a**), 2 µm (**b**) and 10 µm (**c**).

We used FFN^22^ to first create a base segmentation, minimizing merge errors and then agglomerated it to reduce split errors, while keeping merge errors at an acceptable level (Fig. 4a,b). There is a trade-off between having fewer merge errors but more splits versus having fewer splits but more merge errors^22^. Since split errors are easier to resolve in subsequent proofreading steps than merge errors, we have given preference to obtaining low numbers of merge errors throughout training.

**Extended Data Fig. 5:**
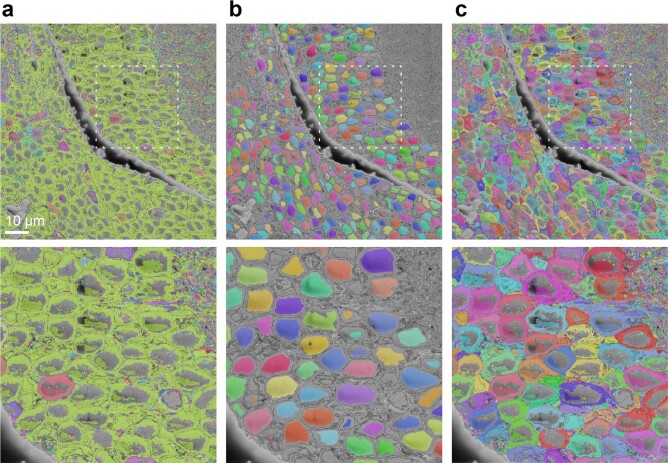
Splitting of merged somata with nuclei segmentation. **a**. Initial segmentation with prolific soma-soma mergers, **b**. Nucleus segmentation, **c**. Initial segmentation split with nucleus segmentation in (**b**).

We trained the FFNs using both manually painted segments and segments generated by proofreading the output of a previously trained FFN. We evaluated the performance of network weight snapshots (‘checkpoints’) saved during training by calculating edge accuracies and merge rates from densely skeletonized subvolumes and from point pairs lying in different but adjacent neurites in the tectum, pretectum and tegmentum. After generating the base segmentation in this fashion, we used FFN resegmentation to compute scores between pairs of adjacent segments^22^, while enforcing separation of the automatically detected cell nuclei and corresponding somata (Extended Data Fig. 5).

We stored the resulting agglomeration graph as a proposal segmentation on a custom backend server and extended the Knossos 3D annotation tool^33^ (https://knossos.app) to provide a live proofreading environment, in which edits are immediately visible by all users. Users start the proofreading procedure by selecting a location within a neuron of interest. Knossos then loads the supervoxel at that location, along with all of the supervoxels that are directly and indirectly connected in the agglomeration graph. The resulting cell, or fragment of a cell, is shown both in a 3D view as well as in slice views overlaid over the raw data. Split and merge errors are corrected interactively by adding or removing connections between supervoxels. In a sample of tectal neurons (*n* = 18), the correction of merge and split errors required on average 2.8 and 98.5 interactions (mouse clicks) per cell, respectively (see Fig. 4c for an illustration of splits).

To estimate the efficiency gain of segmentation proofreading over manual skeletonization, we proofread a random selection of pretectal cells, which had previously been traced manually (*n* = 8). While manual tracing on average proceeded at 0.13 ± 0.04 (mean ± s.d.) mm h^−1^ and proofreading proceeded at 1.11 ± 0.25 mm h^−1^ (average speed-up factor: 9.16 ± 3.16), manual skeletonization requires multiple independent redundant tracings to achieve good reconstruction quality^13,33^. Since we used three to five redundant manual annotators per cell, and since merge errors are easier to commit while skeletonizing, the net manual tracing speed was 0.03 ± 0.01 mm h^−1^, with an average speed-up factor of 60.3 ± 47.9-fold. This corresponded to a total manual skeletonization time for those cells of 57.9 ± 36.5 hours per cell, compared to a proofreading time of 1.09 ± 0.37 hours per cell. A tutorial guiding the user through all steps of the procedure is available at http://mapzebrain.org.

### Synaptic partners of tectal SINs

**Fig. 5:**
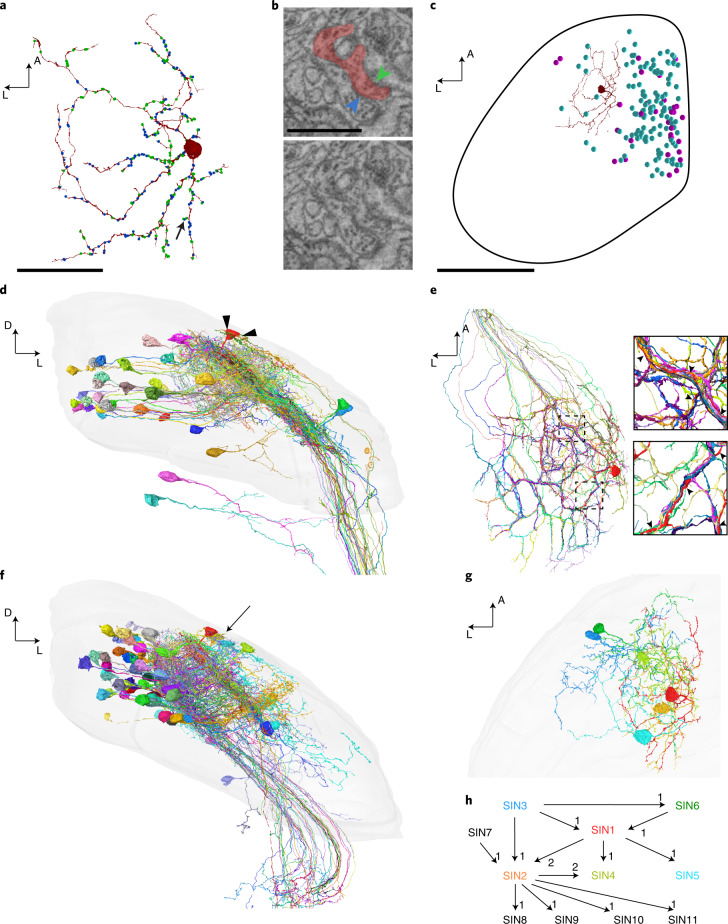
Reconstruction of a SIN and its partners. **a**, Dorsal view of the selected SIN1. Input (blue) and output (green) synapses are indicated. **b**, Annotated (upper panel) and raw data (lower panel) for closely neighboring input (blue) and output (green arrowhead) synapses on a SIN’s neurite (red). Arrow in **a** indicates synapse location. **c**, Dorsal view of left tectum showing cell body locations of all input (purple) and output (cyan) neurons in the anterior tectum. **d**, Frontal view showing the SIN (red) and its presynaptic partners (Supplementary Video 1). Arrowheads indicate RGC input synapses onto SIN cell body. Surface of tectal hemisphere is shown in gray. **e**, Dorsal view of SIN (red) and its input RGC axons in the SFGS layer. Close-ups on the right show that SIN neurites (arrowheads) closely follow the network of RGC axon bundles. **f**, The SIN (red) and its postsynaptic partners (PVINs not shown for clarity, see Supplementary Video 2 for all postsynaptic cells). The second SIN2 for which we mapped all partners is marked by an arrow. **g**, Dorsal view showing a network of interconnected SINs. **h**, Wiring diagram for proofread SINs. Colors match cells shown in **g** (SINs 7–11 are not visualized for clarity). Synapse numbers are indicated next to arrowheads. Scale bars, 35 µm (**a**), 1 µm (**b**) and 85 µm (**c**). A: anterior, L: lateral, D: dorsal.

**Extended Data Fig. 6:**
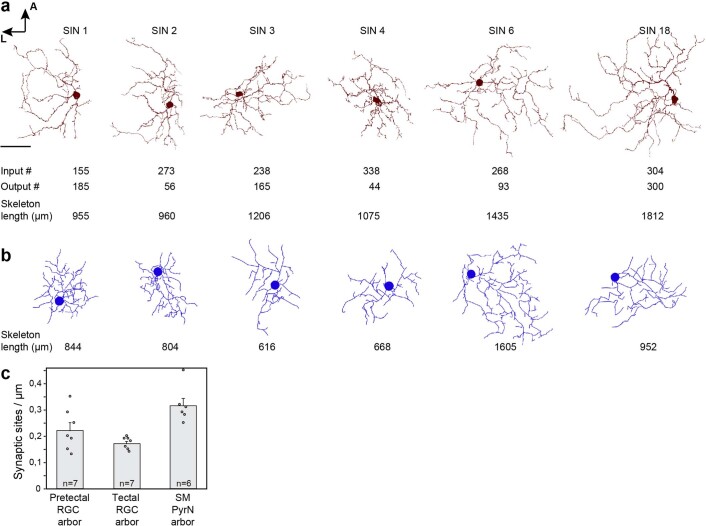
Synapse density and neurite path length comparisons between LM and EM. **a**-**b**. Comparison of reconstructed SINs from our EM dataset (**a**) and from our LM mapzebrain atlas (**b**). The number of input and output synapses is shown below each cell in (**a**) and the neurite path length in µm in (**a**) and (**b**). Scale bar: 30 µm. **c**. Quantification of synapse densities for presynaptic sites of RGCs in the pretectal AF8 and tectal AF10, and for postsynaptic sites of tectal pyramidal neurons (PyrN) in the stratum marginale (SM). Number of cells (n) is provided in each bar. Error bars are SEM.

To benchmark the proofreading workflow, we first traced one neuron, randomly selected among the SINs in the tectum, together with all its presynaptic and postsynaptic partners (SIN1; Fig. 5). SINs are a diverse class of tectal cells that are broadly involved in processing visual stimuli^34–40^. The resulting reconstruction revealed the typical SIN morphology: one extensive, monostratified arborization in the stratum fibrosum et griseum superficiale (layer SFGS3/4, Fig. 5a). We proofread a random selection of other SINs and compared them to all of the LM reconstructions of SINs available in the mapzebrain atlas (Extended Data Fig. 6). Inspection of their neurite path lengths showed a close match, although EM reconstructions resulted in higher details for fine branches and thus in overall longer neurite lengths (EM SINs 1,240.5 ± 332.5 µm; LM SINs 914.6 ± 359.2 µm; mean ± s.d. *n* = 6).

We searched for presynaptic and postsynaptic sites along all the branches of SIN1 (Fig. 5a). Overall we identified 340 synapses (inputs: 155, outputs: 185). Some SIN neurites had a mixed axonal and dendritic character, with presynaptic and postsynaptic sites often in close proximity (Fig. 5a,b). Starting at these sites, we traced all synaptic partners (Fig. 5c–g). Proofreading took on average 9 min until a partner cell type was unambiguously identified. We backtraced axons from retinal ganglion cells (RGCs), whose somata reside in the retina, into the optic tract to determine whether they originated from the same or different RGCs. We identified 75 presynaptic neurons, each forming between 1 and 13 synapses with this particular SIN. These include 41 RGCs, 30 periventricular interneurons (PVINs), two SINs and two cells in the dorsal thalamus (Fig. 5d,e and Supplementary Video 1). A single RGC axon often made multiple (up to 13) synaptic contacts, both locally and on distant dendrites. The 133 postsynaptic cells included 68 PVINs, 46 periventricular projection neurons (PVPNs), four cells with cell bodies in deeper neuropil layers and three other SINs (Fig. 5f,g and Supplementary Video 2). We also observed axo-axonic synapses onto axons of ten RGCs. We found tectal partner cells in the vicinity of the SIN’s arborization, indicating that the SIN network retains retinotopic information (Fig. 5c–g). Presynaptic cells rarely overlapped with postsynaptic cells (three PVINs were both presynaptic and postsynaptic to the SIN), suggesting an overall feedforward circuit organization.

Next we traced all partner cells of another SIN (SIN2) (orange in Fig. 5f–h). SIN2 arborized in the same tectal sublamina and had a similar morphology to SIN1. Input synapses came from RGCs (92), PVINs (45), hindbrain (five), pretectum (three) and other SINs (three, among them SIN1). Of the 47 postsynaptic cells, we identified 18 PVINs, 17 PVPNs, five SINs, two neuropil layers and again axo-axonic synapses onto four RGC axons. Although SIN1 and SIN2 were located in close proximity to each other, with their arbors largely overlapping, they only shared about 15% of input and 21% of output partners. In summary, this effort delivered a network of SINs and all of their synaptic partners.

### A digital address book for all neurons and their connections

**Fig. 6:**
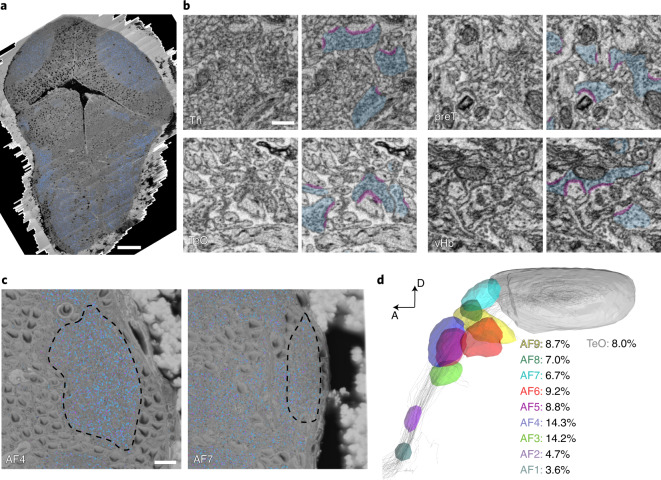
Automatic detection of synaptic contacts. **a**, Dorsal view of the vEM dataset, vesicle clouds labeled in blue. **b**, Close-up examples of automatically segmented vesicle clouds (blue) and synaptic clefts (magenta) in thalamus (Th), pretectum (preT), optic tectum (TeO) and ventral hindbrain (vHb). **c**. Outline of RGC AFs 4 (left) and 7 (right). **d**, Volume fraction occupied by vesicle clouds, calculated for all AFs as a percentage of total area volume. Visualization adapted from mapzebrain atlas. Scale bars, 50 µm (**a**), 500 nm (**b**) and 10 µm (**c**).

Next we automated the detection of synaptic features. For this task, we used the SyConn v.2 pipeline^41,42^. We first used a deep neural network to map synaptic clefts and vesicle clouds (Fig. 6a,b). To validate the quality of synapse annotation, we used ground-truth test data from different parts of the brain, yielding object-wise precision-recall scores close to 1 for synaptic vesicle clouds (tectum 0.98 and 0.96, pretectum 0.98 and 0.96, thalamus 0.91 and 0.98, and ventral hindbrain 0.90 and 0.98 precision and recall, respectively) and synaptic clefts (tectum 0.93 and 0.93, pretectum 0.96 and 0.96, thalamus 0.88 and 0.98, and ventral hindbrain 0.90 and 0.96, respectively). Notably, the volume fraction occupied by synaptic vesicle clouds varied among different neuropil regions. We measured the proportion of synaptic vesicle clouds in the ten retinorecipient arborization fields, including the tectum^32^ (AFs, Fig. 6c,d) and found that the density of synapses was highest in the thalamic neuropil regions AF3 and AF4.

**Extended Data Fig. 7:**
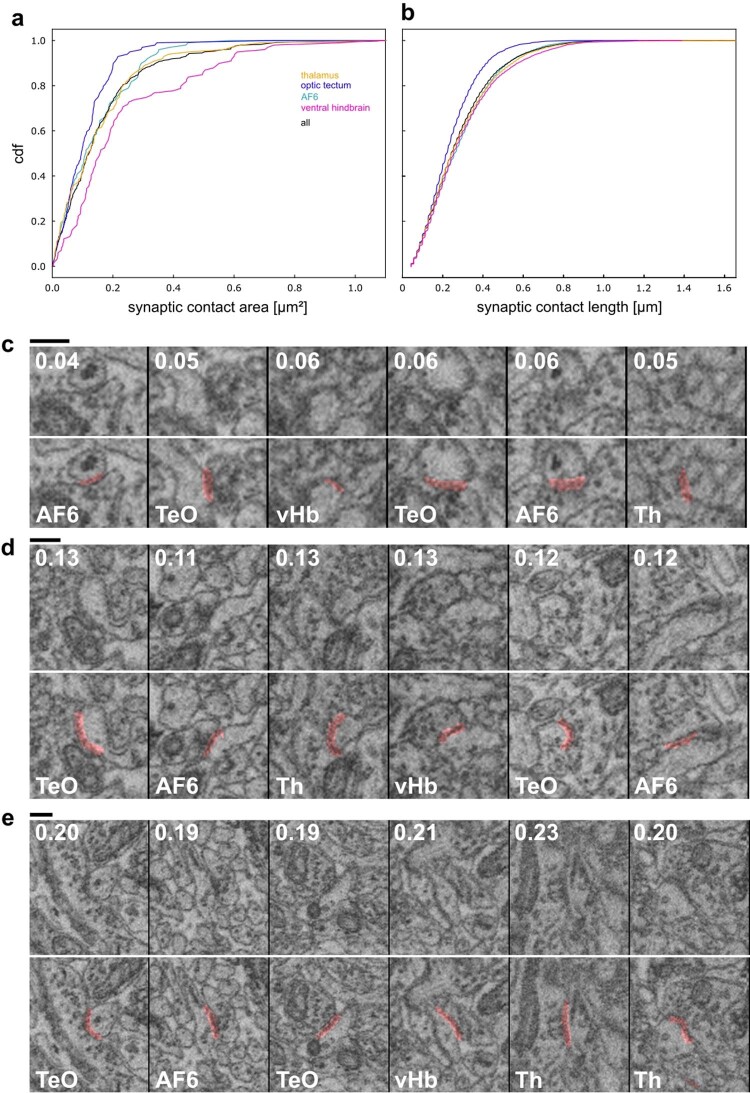
Size of synaptic contacts. **a**. Distribution of synaptic contact areas in different brain areas (n = 100 in each area). **b**. For the same synaptic contacts as in a, distribution of contact lengths as exposed in each intersecting slice. **c**. Examples of small synaptic contacts (20th to 30th percentile by area). Contact area in μm² indicated at the top, brain area at the bottom. **d**, **e**. Like **c**, for intermediate (45th to 55th percentile) and large (70th to 80th percentile) synapses, respectively. Scale bars: 250 nm. AF6: Retinal arborization field 6, TeO: optic tectum, vHb: ventral hindbrain, Th: thalamus.

We quantified synaptic contact area sizes on a sample of 100 synapses each in the thalamus, optic tectum, ventral hindbrain and AF6, finding a median area of 0.12, 0.10, 0.18 and 0.11 µm^2^, respectively (corresponding to median contact lengths over all sections of 243, 220, 280 and 257 nm; Extended Data Fig. 7 and Supplementary Videos 3–5). These values in the larva are lower than those reported for adult zebrafish^43^. We found axonal contact onto the Mauthner cells’ ventral dendrites that were ultrastructurally distinct from chemical synapses, with an intense darkening of the axonal and dendritic membranes interrupted by stretches at which the two membranes visually appeared as if fused into a single membrane (Supplementary Video 6). Since this region of the Mauthner cell receives numerous electrical synapses^44^, some electrical synapses may be identifiable in our dataset.

### Validation of SyConn-based automated synapse detection

SyConn recognized all synapses that a human expert (D.F.) had found in the SINs investigated above, and vice versa, but substantially faster. The SyConn graphical interface allows users to interactively move from cell to cell, tracing a chain of synaptic connections. Using such computer-assisted circuit analysis, we discovered that, in a network of 11 proofread SINs (Fig. 5g,h), synaptic connectivity is unidirectional without apparent recurrency.

Next, we compared the number of automatically identified synapses for specific cell types from our dataset with previously published results from light-microscopic analyses^45,46^. Both for presynaptic sites on RGC axons (0.16 ± 0.02 and 0.13 ± 0.02 mean ± s.e.m. Synaptophysin-labeled puncta per µm neurite path length for pretectal and tectal arborizations, respectively^45^), and for postsynaptic sites on pyramidal cells in the stratum marginale of the tectum (0.4 ± 0.19 s.e.m. PSD95-labeled puncta per micrometer of neurite path length^46^), we obtained similar densities from our vEM dataset (0.22 ± 0.03 and 0.17 ± 0.01 s.e.m. for pretectal and tectal RGC arborizations, respectively, and 0.32 ± 0.03 s.e.m. for pyramidal cells; Extended Data Fig. 6).

**Extended Data Fig. 8:**
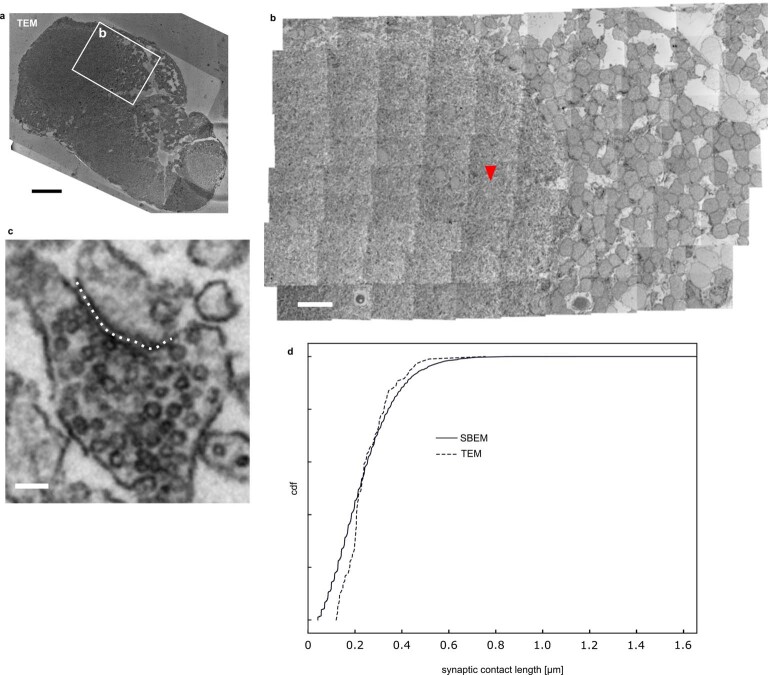
Comparison of synaptic contact lengths in TEM image of optic tectum. **a**. Overview of 35 nm section of a 5 dpf larval zebrafish prepared identically to the sample used for SBEM. **b**. Close-up tile mosaic in optic tectum neuropil. Arrowhead points at synapse in (**c**). **c**. Example synapse. Dotted line illustrates synaptic contact length measurement. **d**. Distribution of contact lengths (n = 100, randomly sampled) in this TEM image (dotted line) compared to the distribution obtained from the optic tectum in the SBEM dataset (as in Extended Data Fig. 7b). Scale bars: a. 50 μm, b. 10 μm, c. 100 nm.

Finally, to examine whether we might have missed some chemical synaptic contacts in this dataset, we acquired a transmission EM image of tectal neuropil from a larval zebrafish brain sample prepared identically to the one used for SBEM at a pixel size of 4.1 by 4.1 nm. We then measured the chemical synaptic contact lengths for a sample of 100 synapses (Extended Data Fig. 8). The good agreement with the size distribution of tectal synapses in the SBEM dataset (Extended Data Figs. 7 and 8) suggests that a large fraction of synapses can indeed be identified. In summary, the synaptic resolution of our dataset combined with our automatic SyConn pipeline allows detection, annotation and quantification of chemical synapses with high confidence.

## Discussion

Here we have described a dataset that contains an EM volume of the larval zebrafish brain, augmented by automatically generated maps of 121,000 nuclei, by synaptic contact locations and by a proposal segmentation of neurites. The resolution is suitable for synapse-scale reconstruction. The EM volume of roughly 0.058 mm^3^ is represented by 12.5 teravoxels (almost 29,000 sections, each 25 nm thick, imaged at 14 × 14 nm^2^ pixel size). The computational tools we developed enable direct extraction of wiring information from the dataset from these data.

We paid attention to the usability of this resource by the community: (1) segmented and proofread data are being made accessible at http://mapzebrain.org and will be continually updated. (2) Open-source software tools (Knossos and SyConn) allow the user to navigate and query the dataset, as well as to contribute their own tracing data to generating a brain-wide connectome. (3) vEM volume and coordinates of the LM atlas of the zebrafish brain^15^ have been coregistered, offering access to additional data modalities for circuit analysis, such as gene and transgene expression, single-cell morphologies and neuroanatomical annotations^47^. Together, these features should lower the entry barrier to connectomic analyses in this system.

Our initial use of this resource has already offered a glimpse into the wealth of biology that awaits discovery. First, we could match 208 cells in the EM volume to functionally characterized neurons in the optic-flow processing pretectum^27^. An in-depth analysis of the wiring diagram of these cells is underway (F.S., W.D., H.B., F.K. in preparation). Second, we revealed the heretofore elusive connectivity of SINs, a largely inhibitory and diverse class of tectal neurons in the tectum, which have been likened to retinal amacrine cells^36^ and our results confirmed the proposed mechanism of feedforward filtering of visual inputs by the SINs^37,39^.

In summary, the new dataset is immediately accessible for circuit interrogations not attainable by LM methods or electrophysiology. Additional demands on this resource are likely to drive further improvements of the computational pipeline and accelerate discoveries. For example, inhibitory and excitatory synapses differ in the shapes of presynaptic vesicles and the thickness of postsynaptic densities^48,49^. Once labeled training data are available, the computational pipeline might be extended to automatically classify synapses by type of transmission^50^. Both electrical connections as well as modulators play important roles in the activity of neural circuits^2^, and explaining their role in the activity of circuits reconstructed from our SBEM dataset will have to be left to subsequent experiments. Combinations of high-resolution EM imaging with LM-based maps of antibody staining patterns in the same sample are likely to prove useful in generating vEM datasets augmented with information on modulator identity in the future^51^. Another direction worth pursuing in the zebrafish system is development and growth. While the present dataset encompasses a larval brain, even the hundredfold larger brains of the adult zebrafish remain amenable for whole-brain EM reconstructions. We expect that technological advances^52^ will make generating connectomes in zebrafish, including in mutants and disease models, a routine task in the future.

## Methods

### Animal husbandry

All animal procedures conformed to the guidelines of the Max Planck Society and the Regierung Oberbayern (protocol number 55.2-1-54-2532-101-12).

### 2P Ca^2+^ imaging

We performed Ca^2+^ imaging in *Tg(elavl3:GCaMP5G)a4598* zebrafish larvae expressing GCaMP5G in almost all neurons at 5 dpf. A few hours before imaging, we fed the larvae paramecia and used only those that consumed paramecia for subsequent imaging. Before imaging, we paralyzed larvae by injecting α-bungarotoxin intraspinally (2 mg ml^−1^ alpha-bungarotoxin (Invitrogen, B1601), FastRed 10% v/v, 1× Danieau’s solution). We used a movable objective microscope (Sutter Instruments) with a Ti:sapphire (Ti:Sa) laser (Chameleon Ultra II, Coherent) to record GCaMP signals (920 nm; roughly 10 mW after the objective) with a ×20 objective (Olympus, numerical aperture 1.0) and used ScanImage software^53^ for image acquisition. We presented visual stimuli to the fish using a custom-built red LED arena (four flat panels covering 360° around the fish; no grating presentation in 30° in front of the fish corresponding to the binocular field). The visual stimulus consisted of vertically oriented gratings moving horizontally in eight phases (gratings in motion for 6 s at spatial frequency of 0.033 cycles per degree and temporal frequency of 2 cycles per s, interspersed with 4 s stationary gratings). Four of the eight phases were monocular, and four were binocular: (1) left nasalward, (2) left temporalward, (3) right temporalward, (4) right nasalward, (5) backward, (6) forward, (7) clockwise and (8) counterclockwise. The sequence of eight phases repeated three times. We recorded a volume centered around the pretectum with roughly 15 *z*-planes separated by 5 µm. The videos in each *z*-plane had a size of 512 × 512 pixels (pixel size of 0.385 µm) at a frame rate of 1.74 Hz.

### Analysis of 2P Ca^2+^-imaging data

We processed GCaMP5G signals with a custom-made routine written in MATLAB^25^. Briefly, we focused on 11 most frequent response types in the pretectum and generated a map of correlated pixels for each corresponding regressor. From these regressor maps, we drew regions of interest to detect correlated cells. We cross-checked each of the identified cells with all the regressor maps for overlap. If the same cell was detected in multiple regressor maps, we assigned it the regressor that gave the highest correlation coefficient value.

### EM sample preparation

After 2P microscopy, we anesthetized the animal in 0.016% tricaine in Ringer solution modified for extracellular space preservation (63 mM NaCl, 63 mM cesium gluconate, 2.5 mM KCl, 25 mM NaHCO_3_, 1.25 mM NaH_2_PO_4_, 25 mM glucose, 2 mM CaCl_2_ and 1 mM MgCl_2_)^17^, based on the principle of extracellular space preservation by cell-impermeable solutes^54^. We removed the eyes with a piece of lasso-shaped tungsten wire and removed the skin covering the brain dorsally by first making a small incision caudal and dorsal of the brain using the electrochemically etched tip of a tungsten wire and then, through this incision, inserting the tungsten wire tip under the skin and carefully pulling upward and rostrally, pulling away the skin without touching the brain. We carefully removed any remaining skin flaps with forceps. Finally, we chemically fixed the larva with 2% glutaraldehyde and stained it with the reduced osmium/thiocarbohydrazide/osmium stain, aqueous uranyl acetate and lead aspartate^8^.

To make the sample sufficiently conductive to allow imaging even the superficial, plastic-adjacent areas of the brain, we dispersed Carbon Black (2.5% w/v, Ketjenblack, AkzoNobel) in the epoxy^55^.

### SBEM data acquisition

**Extended Data Fig. 9:**
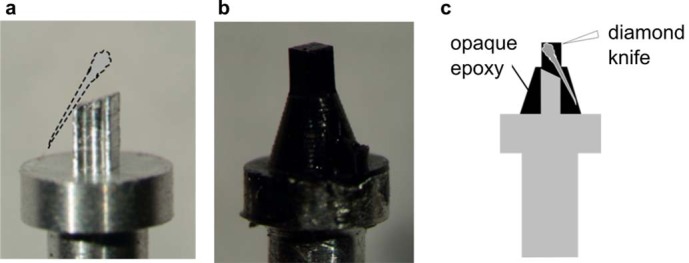
Custom sample holder for reproducible larval zebrafish positioning. **a**. Aluminum stub for fish embedding, **b**. Carbon black epoxy, including fish, cast on the sample holder, **c**. Schematic cross-section.

We embedded the sample using a custom-designed mold and holder that held the sample in a reproducible position and orientation (Extended Data Fig. 9). We then performed X-ray micro-CT imaging of the sample embedded onto the custom holder at 4 µm voxel edge length (SCANCO Medical AG). To generate the EM tile mosaic, we manually segmented the brain tissue in the micro-CT dataset and fit a transformation from the micro-CT coordinate system into that of the SBEM microtome stage motors. By performing dynamic, precomputed X-ray targeted tiling, only 58% of the cuboid bounding box of the brain was scanned.

We performed SBEM acquisition on a Zeiss Ultra Plus scanning electron microscope equipped with a Fibics scan generator. Piezo scanning was used, that is the SBEM stage was smoothly moved along the long axis of the scan, while the electron beam scanned the short axis. This resulted in a tile pattern consisting of elongated images (ranging from 4,615 to 25,000 pixels in length, with an average of 16,206 pixels) stacked horizontally, covering the entire brain with the exception of the retinae (Fig. 1d,e). The ability to extend the FOV to cover the entire brain along one axis reduced the number of FOV positioning moves from 1.3 × 10^6^ to 235,581, saving 46 days in ringdown time, similar to the method described previously^56^. In addition to the piezo-scanned image tiles, a single, low-resolution (200 × 200 nm^2^) overview image was captured per slice with usual electron beam scanning. The microscope was operated by scripted control of the Zeiss SmartSEM v.5 and Fibics ATLAS v.4 software.

We used a GV10x downstream asher (ibss Group, Inc.) to clear the detector diode every 2–3 days. To ensure that no sections were lost due to potential thermic shifts in sample position caused by the asher, we retracted and reapproached the sample every time the downstream asher was activated.

To map the individual image tiles into a single, consistent 3D space, we used the Aligner package^57^ (https://github.com/billkarsh/Alignment_Projects), which optimizes an affine transformation for each image tile. To allow for greater flexibility in the alignment, we cut the individual image tiles into shorter pieces of 500 pixels, with 44 pixels overlap, in length along the piezo-scanned (*y*) axis before registration.

### Alignment of vEM and 2P image stacks

To unambiguously match corresponding neurons between the EM and 2P stacks, we devised the sequential transformation steps as follows. First, we manually identified unique landmark points that were visible in both stacks, such as blood vessel branch points, which we subsequently used for calculating transformations using the BigWarp plugin in Fiji^58^. We identified a total of 1,623 corresponding points representing individual somata in the region where the imaged optic-flow responsive cells were located. We divided the pretectal region into smaller regions (‘blocks’) of size 42 × 28 × 2 pixels and the transformation for each block was calculated separately, based on corresponding points located in a surround of that block. Such local affine transformations were calculated for and applied to 3,426 individual blocks on the left side and 3,676 blocks on the right side of the brain.

### Manual neuron reconstruction and synapse identification

Professional annotators of the neuron reconstruction service ariadne.ai ag (https://www.ariadne.ai) manually reconstructed the neurons and annotated the synapses. Starting from seed points in somata, the annotators reconstructed the skeleton of the neurons by following their neurites within the 3D EM volume using the open-source neuron reconstruction tools Knossos^33^ (https://knossos.app) and PyKNOSSOS^13^ (https://github.com/adwanner/PyKNOSSOS). The annotators manually identified and tagged neurite branch points to revisit and extend them at a later point. We generated consensus skeletons from 3–5 independent reconstructions^13^. The annotators labeled synapses manually using PyKNOSSOS in ‘flight’ mode^13^, where the EM data are displayed in a virtual reslice perpendicular to the local direction of the neurite. They followed the skeletonized axon of every neuron along precalculated paths and annotated all output synapses as described previously^11^. In brief, a synapse was inferred when (1) axonal and dendritic neurite membrane surfaces were parallel and directly apposed, (2) a vesicle cloud was seen in the axon with vesicles in close proximity to the axonal membrane opposite of the dendrite and (3) a thickening or darkening, potentially very faint, of the postsynaptic neurite membrane was observed. In addition, the annotators assigned a subjective confidence level to each synapse. Two independent human annotators performed synapse annotation. If the postsynaptic partner was among the reconstructed cells, this was labeled as such. If a synapse location was found by only one of the two independent annotators, a third expert annotator (F.S.) made the final decision.

We measured neurite diameters in the set of skeletonized cells by randomly sampling 200 locations from axons and dendrites each, and keeping fragments of the skeleton within a radius of 750 nm around these locations. The fragments were loaded into PyKNOSSOS and displayed in ‘flight’ mode, allowing a local measurement of the neurite diameter.

### Morphological characterization of pretectal neurons

We compared the targeting of anatomical areas between simple and complex pretectal cells by counting for every cell the number of branch points intersecting one of the region annotations from the light-level atlas registered to our EM dataset. We used branch points instead of all skeleton points to not count parts of the axons and dendrites that only pass through a region. We normalized the branch point counts to the total number of branch points in each category.

### Synapse size measurement

We measured synapse sizes in our SBEM dataset by taking a random sample of 100 synaptic contact locations automatically detected in each one of AF6, optic tectum, thalamus and ventral hindbrain, as defined by the mapzebrain region annotations mapped to the SBEM dataset. We manually reviewed these 400 locations to make sure that they precisely represented exactly one synaptic contact and corrected them if necessary. We then calculated contact areas from surface meshes of those contact annotations generated by the zmesh python library (https://github.com/seung-lab/zmesh). To enable a comparison to data derived from single two-dimensional (2D) sections, we sliced the 3D contact area objects along each one of the three cardinal directions, spaced by the resolution of the dataset and measured the length of the 2D profile exposed in each section.

For high-resolution synapse size measurement, we used a single 35-nm slice of a sample prepared identically to the one used for our SBEM dataset, which we imaged on a JEOL JEM-1230 transmission electron microscope, equipped with a Gatan Orius SC1000 digital camera, at 12,000-fold magnification (pixel size 4.1 × 4.1 nm^2^) at 80 kV. We measured synaptic contact lengths by sampling 1.5 × 1.5 µm^2^ subregions of the tectal neuropil randomly and annotating all intersecting synaptic contacts, until 100 synapses had been measured.

### Registration to standard brain

We trained a 2D U-Net^59^ to distinguish soma and neuropil regions in the vEM dataset (Fig. 3b). In parallel, we obtained a soma and neuropil map in the standard brain coordinate system by thresholding and summing the *elavl3:H2B-GCaMP6s* (for cell nuclei) and the *elavl3:lynTag-RFP* (for neuropil) reference brain channels (Fig. 3a). This allowed us to calculate a diffeomorphic transformation to map these two datasets using the dipy^60^ (https://dipy.org/) registration toolkit, which compensates for the complex deformations that the sample underwent during EM preparation.

### Tissue classification

Tissue classification models 1 and 2 used the architecture and training hyperparameters described in previous work^22^. Model 1 operated on 28 × 28 × 25 nm^3^ data and was trained on manually annotated labels of neuropil (35 megavoxels, MVx), soma (13 MVx) and a class containing blood vessels and ventricles (4 MVx). Model 2 operated on 56 × 56 × 100 nm^3^ data and was trained on manually annotated labels of neuropil (114 MVx) and nonneuropil (33 MVx). The annotations for both networks were made independently. Model 3 operated on 56 × 56 × 100 nm^3^ data and was trained on binary labels of nucleus (20 MVx) versus not-nucleus (15 MVx). The network architecture was a stack of 16 3D valid-mode convolutions with 32 feature maps, ReLU activation and additive skip connections around every two convolutions, with the exception of the first two. The output of the convolution stack was processed by a point-wise convolution with two feature maps representing the class logits. We trained this network using cross-entropy loss with asynchronous stochastic gradient descent at a learning rate of 10^−3^, batch size of 16 and eight NVIDIA V100 workers. Examples were sampled with equal frequency from every class during training.

We binarized the results of model 3, set any voxels predicted as ‘nonneuropil’ or ‘soma’ by model 1 or 2 to 0, and computed the 3D connected components of the results to form an initial soma segmentation. We then applied morphological erosion with radius of 5 to reduce false mergers, and recomputed the 3D connected components. Objects with a volume of more than 1,000 pixels (corresponding to the volume of a sphere with a radius of around 700 nm) were retained as automatically detected soma candidates.

We detected defocused regions by filtering the in-plane CLAHE-normalized images with a Gaussian with sigma of 1, followed by a discrete Laplacian filter. For every voxel, we computed the standard deviation of the filtered image within a 21 × 21 region centered at that voxel. We downsampled the results 128× in-plane with area-averaging, and labeled voxels with values <57 as defocused.

### Neurite segmentation

We trained FFNs using three types of ground truth: (1) manually painted segments (three MVx spread over three subvolumes), (2) manually corrected automated segmentation (15 MVx) and (3) manually agglomerated neurites from a previous automated segmentation (59 neurites containing 206 MVx). The segmentations used for manual agglomeration and merge error proofreading were created with an earlier FFN model trained only with the manually painted ground truth.

During network training, we sampled from the three types of ground truth at relative frequencies of 0.85, 0.05 and 0.1. Within a given type of ground truth, training examples were selected as previously described^22^.

We used all network weight snapshots (‘checkpoints’) saved during training to segment three 5 × 5 × 6.5 μm^3^ subvolumes for which dense skeleton tracings were previously traced by human annotators. These skeleton tracings allowed us to compute the average edge accuracy^22^ for every network checkpoint. We selected the seven checkpoints with the highest accuracies, and used them to segment the complete volume with forward and reverse seed ordering. During segmentation, we used on-the-fly realignment with a maximum translation limit of 10 px to correct for local alignment problems in the EM imagery, as well as FFN FOV movement restriction when postrealignment section-to-section offset exceeded 4 px. We also restricted the FFN FOV center from moving to any voxel predicted to belong to a blood vessel, ventricle or not-neuropil by at least one of the tissue classification models, or when estimated to have insufficient focus. We computed the oversegmentation consensus between the forward and reverse seed orderings^22^ for every checkpoint and used a set of 217 manually annotated point pairs within the tectum, pretectum and tegmentum to screen the seven resulting segmentations for merge errors. The points were located so that each element of the pair lay within a different but adjacent neurite. For every segmentation, we computed the number of point pairs for which the corresponding voxels were labeled with two distinct nonzero segment IDs. To form the base segmentation we then selected the two segmentations that together correctly labeled the highest number of these point pairs, computed their oversegmentation consensus and postprocessed it by removing unlabeled voxels in the interior of segments, as well as merging segments completely contained within other segments^61^.

### Semiautomatic neuron reconstruction (proofreading)

The Knossos-based proofreading tool makes available a live proofreading environment in which edits contributed by different users are immediately available to all users. It interacts with a custom backend server, which manages the agglomeration graph. Knossos can (1) request a list of objects that are part of the same agglomeration graph connected component, (2) add a connection, (3) remove a connection between two objects in the agglomeration graph and (4) request the surface mesh representations for a set of objects. For efficiency, the backend server holds the agglomeration graph in random access memory. Any edit to the agglomeration graph is directly performed in memory, but also written to an on-disk append-only edit log. The edit log is replayed on top of the original automatically generated proposal agglomeration every time the server is reloaded. This design allows highly efficient operations: in our usage so far, edge insertions required 4, 20 and 42 ms (first percentile, median, 99th percentile, respectively, with 13,461 insertions), edge deletions required 1, 18 and 39 ms (1,875 deletions) and obtaining the connected component to which a supervoxel belonged by depth-first search required 0, 12 and 111 ms (40,311 requests).

**Extended Data Fig. 10:**
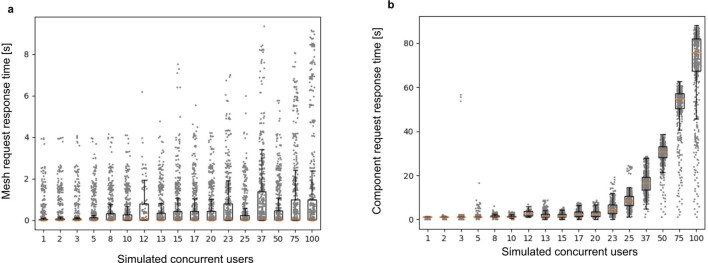
Simulation of proofreading server performance under different concurrent user loads. **a**. Response time to request of meshes for cells (either complete or currently undergoing proofreading) and (**b**) to the request of agglomeration graph connected component containing a given supervoxel, depending on the number of concurrently active simulated users. Box plot center lines represent medians, box limits upper and lower quartiles, whiskers 1.5x the interquartile range.

We performed a stress-test of the backend server by replaying real user activity from log files with increasing parallelization, from a single client machine connected to the backend server by a 1 gigabit ethernet connection (Extended Data Fig. 10).

To identify all synaptic partners of SINs in the tectum, we first manually annotated all incoming and outgoing synapses along the SIN neurites. Starting from these synapses, we then traced the partner cells, until the cell body or, for RGCs, the axon in the optic tract were unambiguously identified. In case of tectal cells, presence of an axon leaving the tectum was used as the criterion to distinguish PVPNs from PVINs.

### Automated synapse detection by machine learning

In collaboration with ariadne.ai ag, we trained a 3D multiclass U-Net^59,62^ to predict the locations of synaptic vesicle clouds and synaptic clefts over the entire volume. To ensure optimal prediction quality, we performed multiple rounds of prediction on test regions, followed by the addition of ground truth targeted to mistakes found in these regions. In total, 313.26 MVx of ground truth were used to train the final network. We quantified the quality of the automatic synapse detection by comparing to randomly selected, manually annotated test volumes in the thalamus, tectum, pretectum and ventral hindbrain. A total of ten test volumes, each 3.5 × 3.5 × 3.5 μm^3^ in size, were created, containing a total of 1,375 synaptic clefts and 971 vesicle clouds. To quantify vesicle cloud density in different RGC AFs, we mapped the mapzebrain AF region annotations to the EM data and manually refined them to exclude somata from neuropil regions and calculate the density of voxels predicted to be part of vesicle clouds within these regions.

### Mapping of automatic synapse detection to neurite segmentation

We used SyConn2 (ref. ^42^) to process the automatic synaptic cleft, synaptic vesicle cloud and neurite segmentations and generate a synaptic connectome. The respective processing parameters and a source code snapshot can be found under https://gitlab.mpcdf.mpg.de/pschuber/SyConn/-/tree/chunk_mask.

We then used synapses mapped to proofread cells to automatically extract SIN connectivity, and to compare synapse densities on pyramidal tectal interneurons and RGCs to data available in the literature.

### Statistics and reproducibility

Box plot center lines represent medians, box limits upper and lower quartiles, whiskers 1.5× the interquartile range. Error bars in bar plots represent s.e.m. If not stated otherwise, wherever representative example micrographs are shown, results are derived from a minimum of ten samples. Significance of difference of means was tested with Welch’s *t*-test and Holm–Bonferroni multiple testing correction to a significance level of *P* < 0.05.

### Reporting summary

Further information on research design is available in the Nature Research Reporting Summary linked to this article.

## Online content

Any methods, additional references, Nature Research reporting summaries, source data, extended data, supplementary information, acknowledgements, peer review information; details of author contributions and competing interests; and statements of data and code availability are available at 10.1038/s41592-022-01621-0.

## Supplementary information

Reporting Summary

Supplementary Video 1 3D rendering of SIN input partner cells.

Supplementary Video 2 3D rendering of SIN output partner cells.

Supplementary Video 3 Small (20th to 30th percentile by area) example synapses.

Supplementary Video 4 Intermediate (45th to 55th percentile by area) example synapses.

Supplementary Video 5 Intermediate (70th to 80th percentile by area) example synapses.

Supplementary Video 6 Putative gap junctions on Mauthner cell ventral dendrite.

## Data Availability

The dataset generated during this study, as well as the Max Planck Zebrafish Brain Atlas, are available at http://mapzebrain.org along with detailed instructions for use of the agglomeration graph proofreading tool and download of the associated data.

## Code availability

The Knossos proofreading tool is available from https://github.com/knossos-project/knossos/tree/bmpr, licensed under the GPL v.2. The agglomeration proofreading backend server is available from https://github.com/ariadne-ai/agglomeration_proofreading, licensed under the AGPL v.2.
