# Supplementary material for: Automated synapse-level reconstruction of neural circuits in the larval zebrafish brain
Source: Nat Methods. 2022 Oct 24;19(11):1357–66. doi: 10.1038/s41592-022-01621-0 (PMC9636024; doi:10.1038/s41592-022-01621-0)
Supplement: Supplementary file 1 — Reporting Summary [file 41592_2022_1621_MOESM1_ESM.pdf]

nature research

Corresponding author(s): Herwig Baier

Last updated by author(s): Jun 7, 2022

Reporting Summary

Nature Research wishes to improve the reproducibility of the work that we publish. This form provides structure for consistency and transparency in reporting. For further information on Nature Research policies, see our Editorial Policies and the Editorial Policy Checklist.

Statistics

For all statistical analyses, confirm that the following items are present in the figure legend, table legend, main text, or Methods section.

n/a	Confirmed	
☐	☒	The exact sample size (n) for each experimental group/condition, given as a discrete number and unit of measurement
☐	☒	A statement on whether measurements were taken from distinct samples or whether the same sample was measured repeatedly
☒	☐	The statistical test(s) used AND whether they are one- or two-sided<br>*Only common tests should be described solely by name; describe more complex techniques in the Methods section.*
☒	☐	A description of all covariates tested
☒	☐	A description of any assumptions or corrections, such as tests of normality and adjustment for multiple comparisons
☐	☒	A full description of the statistical parameters including central tendency (e.g. means) or other basic estimates (e.g. regression coefficient) AND variation (e.g. standard deviation) or associated estimates of uncertainty (e.g. confidence intervals)
☒	☐	For null hypothesis testing, the test statistic (e.g. F, t, r) with confidence intervals, effect sizes, degrees of freedom and P value noted<br>*Give P values as exact values whenever suitable.*
☒	☐	For Bayesian analysis, information on the choice of priors and Markov chain Monte Carlo settings
☒	☐	For hierarchical and complex designs, identification of the appropriate level for tests and full reporting of outcomes
☒	☐	Estimates of effect sizes (e.g. Cohen's d, Pearson's r), indicating how they were calculated

Our web collection on statistics for biologists contains articles on many of the points above.

Software and code

Policy information about availability of computer code

Data collection	SBEM Data Acquisition: Zeiss SmartSEM v5 and Fibics ATLAS v4
Data analysis	MATLAB R2014b (MathWorks)<br>Aligner for large scale serial section image data (https://github.com/billkarsh/Alignment_Projects, git version of May 15, 2016)<br>Knossos (https://github.com/knossos-project/knossos/tree/bmpr, git version of December 3, 2021)<br>Flood-Filling Networks (https://github.com/google/ffn; Januszewski et al., Nature Methods 2018)<br>Dipy (https://dipy.org/, v1.0)<br>PyKnossos (https://github.com/adwanner/PyKNOSSOS, git version of December 3, 2021)<br>SyConn2 (https://gitlab.mpcdf.mpg.de/pschuber/SyConn/-/tree/chunk_mask, git version of December 3, 2021)<br>Proofreading server (https://github.com/ariadne-ai/agglomeration_proofreading, git version of March 29, 2022)<br><br>Where "git version of (date)" is specified, the checked out code version from that exact date was used and no exactly corresponding release version number is available.

For manuscripts utilizing custom algorithms or software that are central to the research but not yet described in published literature, software must be made available to editors and reviewers. We strongly encourage code deposition in a community repository (e.g. GitHub). See the Nature Research guidelines for submitting code & software for further information.

Data

Policy information about availability of data

All manuscripts must include a data availability statement. This statement should provide the following information, where applicable:

- Accession codes, unique identifiers, or web links for publicly available datasets
- A list of figures that have associated raw data
- A description of any restrictions on data availability

A tutorial guiding the user through the steps of the procedure and providing links to the SBEM dataset and the software tools is available at https://mapzebrain.org/. Instructions for downloading the data are provided in that tutorial.
The single-cell and region annotation atlas is also available through a web interface at https://mapzebrain.org

Field-specific reporting

Please select the one below that is the best fit for your research. If you are not sure, read the appropriate sections before making your selection.

☒ Life sciences ☐ Behavioural & social sciences ☐ Ecological, evolutionary & environmental sciences

For a reference copy of the document with all sections, see nature.com/documents/nr-reporting-summary-flat.pdf

Life sciences study design

All studies must disclose on these points even when the disclosure is negative.

Sample size	Due to the long duration of volume electron microscopy (vEM) experiments and the experimental challenges involved in correlating 2-photon and vEM data, exactly one zebrafish brain was acquired.
Data exclusions	No data was excluded.
Replication	Since the aim of this work is to provide a complete zebrafish brain at synaptic resolution as a resource to the community, replication is beyond the scope of this study. Future work will hopefully shed light on inter-individual variability.
Randomization	Since the goal of this study was to successfully acquire a correlative 2-photon/SBEM dataset for a single larval zebrafish, no allocation to different groups was performed. To ensure general good health, only larvae were selected that successfully captured prey (Paramecium caudatum). Of the successfully imaged fish, the one with the largest number of stimulus-correlated pretectal cells was used for vEM dataset acquisition.
Blinding	Since exactly one zebrafish brain was acquired, no blinding of group allocation was required.

Reporting for specific materials, systems and methods

We require information from authors about some types of materials, experimental systems and methods used in many studies. Here, indicate whether each material, system or method listed is relevant to your study. If you are not sure if a list item applies to your research, read the appropriate section before selecting a response.

Materials & experimental systems

n/a	Involved in the study	
☒	☐	Antibodies
☒	☐	Eukaryotic cell lines
☒	☐	Palaeontology and archaeology
☐	☒	Animals and other organisms
☒	☐	Human research participants
☒	☐	Clinical data
☒	☐	Dual use research of concern

Methods

n/a	Involved in the study	
☒	☐	ChIP-seq
☒	☐	Flow cytometry
☒	☐	MRI-based neuroimaging

Animals and other organisms

Policy information about studies involving animals; ARRIVE guidelines recommended for reporting animal research

Laboratory animals	The zebrafish (Danio rerio) transgenic line Tg(elavl3:GCaMP5G)a4598 was used at 5 days post fertilization.
Wild animals	No wild animals were used.

Field-collected samples	No field-collected samples were used.
Ethics oversight	The Regierung Oberbayern approved all animal procedures (protocol number 55.2-1-54-2532-101-12).

Note that full information on the approval of the study protocol must also be provided in the manuscript.
